# The complete mitochondrial genome of *Pentatoma rufipes* (Hemiptera, Pentatomidae) and its phylogenetic implications

**DOI:** 10.3897/zookeys.1042.62302

**Published:** 2021-06-08

**Authors:** Ling Zhao, Jiufeng Wei*, Wanqing Zhao, Chao Chen, Xiaoyun Gao, Qing Zhao

**Affiliations:** 1 College of Plant Protection, Shanxi Agricultural University, Taigu 030801, Shanxi, China Shanxi Agricultural University Taigu China; 2 Department of Biology, Xinzhou Teachers University, Xinzhou 034000, Shanxi, China Xinzhou Teachers University Xinzhou China

**Keywords:** Mitogenome, Pentatomoidea, phylogenetic analysis

## Abstract

*Pentatoma rufipes* (Linnaeus, 1758) is an important agroforestry pest widely distributed in the Palaearctic region. In this study, we sequence and annotate the complete mitochondrial genome of *P. rufipes* and reconstruct the phylogenetic trees for Pentatomoidea using existing data for eight families published in the National Center for Biotechnology Information database. The mitogenome of *P. rufipes* is 15,887-bp-long, comprising 13 protein-coding genes, 22 transfer RNA genes, two ribosomal RNA genes, and a control region, with an A+T content of 77.7%. The genome structure, gene order, nucleotide composition, and codon usage of the mitogenome of *P. rufipes* were consistent with those of typical Hemiptera insects. Among the protein-coding genes of Pentatomoidea, the evolutionary rate of ATP8 was the fastest, and COX1 was found to be the most conservative gene in the superfamily. Substitution saturation assessment indicated that neither transition nor transversion substitutions were saturated in the analyzed datasets. Phylogenetic analysis using the Bayesian inference method showed that *P. rufipes* belonged to Pentatomidae. The node support values based on the dataset concatenated from protein-coding and RNA genes were the highest. Our results enrich the mitochondrial genome database of Pentatomoidea and provide a reference for further studies of phylogenetic systematics.

## Introduction

The mitochondrion is a semi-autonomous organelle with its own genetic material, known as the mitochondrial genome (mitogenome) (Nass and Nass 1963). The mitogenome is widely used in the fields of molecular evolution, phylogenetic analysis, molecular ecology, biogeography, and population genetics because of its advantages of small size, stable genetic composition, and maternal inheritance (Ballard and Whitlock 2004; Simon and Hadrys 2013; Cameron 2014; Yuan and Guo 2016). Insects, as the most diverse, numerous, and widely distributed animals on Earth, are hotspots in mitogenome research (Boore 1999). To date, mitochondrial genome research has been very extensive, covering all orders of insects (Cameron 2014). Insect mitogenomes are covalently closed, double-stranded, circular DNA molecules (14–20 k bp long), and usually contain a control region and 37 genes: 13 protein-coding genes (PCGs), 22 transfer RNA (tRNA) genes, and two ribosomal RNA (12S rRNA and 16S rRNA) genes (Boore 1999; Cameron and Whiting 2008; Cameron 2014). The structure of mitogenome in most known insects is stable, and the gene arrangement is relatively conservative, which are consistent with the genome composition and arrangement of the most typical insect mitochondrial genome, namely *Drosophila yakuba* Burla (Clary and Wolstenholme 1985).

Pentatomoidea, one of the most commonly encountered groups in Hemiptera, includes 1,410 genera and 8,042 species which are widely distributed worldwide (Rider et al. 2018). Pentatomoid insects have diverse feeding habits, although the majority are herbivorous. Some cause huge economic losses, such as *Dolycoris baccarum* (Linnaeus) and *Halyomorpha halys* Stål. In addition, some pentatomoid insects are predatory, including most of the species of Asopinae; and a few groups are suspected to be fungus feeders, such as members of the Canopidae and Megarididae (Rider et al. 2018; Zhao et al. 2018). Classification of the superfamily Pentatomoidea has long been contentious; and different scholars have distinct opinions. For example, Schaefer (1993) divided Pentatomoidea into 16 families, whereas Henry (1997) recognized 17 families, placing Eumenotidae and Thyreocoridae at the family level. Grazia et al. (2008) supported the monophyly of Pentatomoidea and most of the included families, which was based on morphological characters and molecular markers (16S rRNA, 18S rRNA, 28S rRNA and COI); Wu et al. (2016) reconstructed the phylogenetic relationships of 16 families within Pentatomoidea using 18S and 28S rDNAs sequences and showed that Cydnidae and Tessaratomidae might be polyphyletic; Lis et al. (2017) combined 28S+18S rDNA sequence, questioned the monophyleticity of the “cydnoid” complex of pentatomoid families (Cydnidae, Parastrachiidae, Thaumastellidae, and Thyreocoridae), and demonstrated the polyphylicity of Cydnidae. Recently, many taxonomists reorganized the families, genera, and species of Pentatomoidea, and divided Pentatomoidea into 18 families (Rider et al. 2018). With the development of next-generation sequencing (NGS), an increasing number of pentatomoid mitogenome sequences have been obtained, which provide the possibility of resolving the phylogenetic relationships among the superfamily at the genetic level (Yuan et al. 2015b; Bai et al. 2018; Zhao et al. 2018). Furthermore, Wu et al. (2017) confirmed the monophyly of Scutelleridae (based on 18S + 28S rDNAs + 13PCGs), and Liu et al. (2019) reconstructed the phylogeny of Pentatomomorpha and Pentatomoidea based on PCGRNA and PCG12RNA. However, despite the abundance of species in the superfamily, only 97 species have complete or nearly complete mitogenomes published in the National Center for Biotechnology Information (NCBI; https://www.ncbi.nlm.nih.gov/2020.07); these represent only eight families. Moreover, there has been no discussion about the phylogenetic position of *Pentatoma* species, except for the description of *Pentatoma semiannulata* (Motschulsky) mitogenome by Wang et al. (2021). Therefore, it is necessary to determine more mitogenome sequences of *Pentatoma* species to better understand its phylogenetic relationships.

*Pentatoma rufipes* (Linnaeus, 1758) (Hemiptera, Heteroptera, Pentatomidae) is a medium-sized to large, dark brown insect with reddish-orange spots and bright orange legs (Hsiao 1977; Bantock and Botting 2013). These insects are widely distributed in the Palearctic region (Ling and Zheng 1987; Fan and Liu 2012). They can damage oak, poplar, elm, hawthorn, apricot, pear, and other trees, and they constitute an important agricultural and forestry pest (Hsiao et al. 1977; Powell 2020). There are also records of *P. rufipes* preying on *Zygaena filipendulae* L.(Lepidoptera, Zygaenidae) (Hamilton and Heath 1976). Previous studies on *P. rufipes* mostly focused on its physiological and morphological characteristics (Ling and Zheng 1987; Neupert et al. 2009), with limited molecular data on the mitochondrial COI and COII genes (Bu et al. 2005; Liang 2009), along with some studies identifying biological characteristics and potential control strategies (Peusens and Beliën 2012; Powell 2020).

In this study, we sequenced and annotated the mitogenome of *P. rufipes* and analyzed its mitogenome in detail, including the genome structure, nucleotide composition, and codon usage, and constructed RNA secondary structures. In addition, we combined the complete mitogenome of *P. rufipes* with the existing data for the eight families of Pentatomoidea to explore the phylogenetic position of *P. rufipes*.

## Materials and methods

### Sample collection

Adult *Pentatoma rufipes* specimens were collected in Baiji Hill (Tonghua City, Jilin Province, China; 41°58.14'N, 126°06.58'E) on 24 July 2015. All samples were immediately placed in absolute ethanol and stored in a freezer at –20 °C until DNA extraction. Specimen identification was performed by Qing Zhao. The voucher specimen is maintained at the Institute of Entomology of Shanxi Agricultural University (voucher number: SXAU 007; Taigu, China). The complete mitogenome of *P. rufipes* has been submitted to GenBank (accession number: MT861131).

### DNA extraction and sequencing

Whole-genome DNA was extracted from the thoracic muscle of adult samples using the Genomic DNA Extraction Kit (Sangon Biotech, Shanghai, China). The mitogenomes were sequenced using the whole-genome shotgun method on the Illumina Miseq platform (Personalbio, Shanghai, China), with 400-bp inserts and paired-end model. A5-miseq v. 20150522 (Coil et al. 2015) and SPAdes v. 3.9 (Bankevich et al. 2012) were used to assemble the data.

### Genome annotation and sequence analysis

After assembly, the complete mitogenome was manually annotated using Geneious v. 8.1.4 software (Kearse et al. 2012). A reference sequence of *Eurydema gebleri* Kolenati for annotation was obtained from the basic local alignment search tool (BLAST) in the NCBI database. The boundaries of the PCGs were determined using Open Reading Frame Finder (http://www.ncbi.nlm.nih.gov/gorf/gorf.html) on the NCBI website. MEGA v. 7.0 (Kumar et al. 2016) was used to translate the proteins to verify the start codons, stop codons, and amino acid sequences and to ensure the accuracy of the sequences. We annotated tRNA sequences using tRNAscan-SE (http://lowelab.ucsc.edu/tRNAscan-SE/) (Lowe and Eddy 1997) and MITOS (http://mitos.bioinf.uni-leipzig.de/index.py/) (Bernt et al. 2013) with the invertebrate mitochondrial code. The boundaries of rRNA genes were completed according to the positions of adjacent genes and published rRNA gene sequences from Pentatomidae insects in GenBank (Boore 2006). The codon usage, base composition, and amino acid composition of the mitogenome were analyzed using MEGA v. 7.0. The skew of the nucleotide composition was calculated with the formulas: AT-skew = (A – T) / (A + T) and GC-skew = (G – C) / (G + C) (Perna and Kocher 1995).

### Phylogenetic analyses

In this study, we selected the mitogenomes of *P. rufipes*, representative species from eight other Pentatomoidea families, and two Coreoidea species (outgroup) to analyze the phylogenetic position of *P. rufipes* and the phylogenetic relationships within Pentatomoidea. All species included in this analysis are listed in Table 1. The nucleic acid sequences of the 13 PCGs were extracted using Geneious v. 8.1.4. All PCGs were translated into their amino acid sequences and aligned using MUSCLE with default parameters in MEGA v. 7.0 (Edgar 2004). The tRNA and rRNA genes were also aligned using the MUSCLE algorithm in MEGA v. 7.0. The resulting alignments were concatenated into a combined matrix.

**Table 1.** List of species used to construct the phylogenetic tree.

**Classificationstatus**	**Family**	**Species**	**Accession number**
**Outgroup**			
Coreoidea	Coreidae	*Hydaropsis longirostris*	EU427337
		*Anoplocnemis curvipes*	NC_035509
**Ingroup**			
Pentatomoidea	Acanthosomatidae	*Acanthosoma labiduroides*	JQ743670
		*Sastragala edessoides*	JQ743676
		*Anaxandra taurina*	NC_042801
	Cydnidae	*Macroscytus gibbulus*	NC_012457
		*Adrisa magna*	NC_042429
		*Scoparipes salvazai*	NC_042800
	Dinidoridae	*Cyclopelta parva*	KY069962
		*Megymenum gracilicorne*	NC_042810
	Pentatomidae	*Halyomorpha halys*	NC_013272
		*Eurydema gebleri*	NC_027489
		*Graphosoma rubrolineatum*	NC_033875
		*Gonopsis affinis*	NC_036745
		*Dinorhynchus dybowskyi*	NC_037724
		*Plautia fimbriata*	NC_042813
		***Pentatoma rufipes***	**MT861131**
	Plataspidae	*Coptosoma bifaria*	EU427334
		*Megacopta cribraria*	NC_015342
	Scutelleridae	*Cantao ocellatus*	NC_042803
		*Eurygaster testudinaria*	NC_042808
	Tessaratomidae	*Dalcantha dilatata*	JQ910981
		*Eusthenes cupreus*	NC_022449
		*Tessaratoma papillosa*	NC_037742
	Urostylididae	*Urostylis flavoannulata*	NC_037747

To determine if the sequences contained phylogenetic information, we tested nucleotide substitution saturation, and plotted transition and transversion substitutions against the TN93 distance for all datasets before reconstructing the phylogenetic trees using DAMBE v. 4.5.32 (Xia and Xie 2001; Xia and Lemey 2009). The optimal substitution models for each dataset were calculated using PartitionFinder v. 1.1.1 (Lanfear et al. 2012). Phylogenetic analyses were conducted using the Bayesian inference method, in MrBayes v. 3.2.5 (Ronquist et al. 2012) under the GTR+G+I substitution model with four independent Markov chains run for 10,000,000 generations and stopped when the average standard deviation value was below 0.01. The first 25% of trees were discarded as burn-ins, and the remaining trees were used to construct a 50% majority-rule consensus tree (Zhao et al. 2018). The phylogenetic trees were constructed using three types of datasets: (1) all codon positions of the 13 PCGs; (2) the 13 PCGs, excluding the third codon position (PCG12); and (3) the PCGs, 22 tRNA genes, and two rRNA genes (PCGRNA).

## Results

### Genomic features

**Figure 1.**
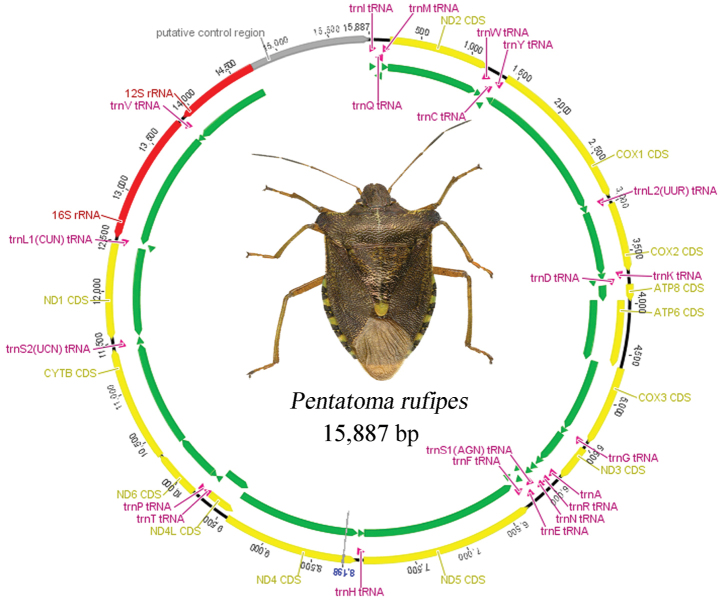
Mitochondrial genome map of *Pentatoma rufipes*. Arrows indicate the orientation of gene transcription. Protein coding and ribosomal genes are shown with standard abbreviations.

The mitochondrial genome of *Pentatoma rufipes* is 15,887-bp-long and contains a control region and 37 genes comprising 13 PCGs, 22 tRNA genes and two rRNA genes (Fig. 1; Table 2). Among these genes, 14 genes are located on the minority strand (N-strand), including four PCGs (ND5, ND4, ND4L, and ND1), eight tRNA genes (trnQ, trnC, trnY, trnF, trnH, trnP, trnL1(CUN), and trnV), and two rRNA genes (12S rRNA and 16S rRNA genes), whereas the remaining 23 genes are encoded on the majority strand (J-strand). The mitogenome is compact, with a total of nine gene overlaps, ranging in length from 1 to 8 bp; the longest overlap is between trnW and trnC. Furthermore, there were 16 gene spacers from 1 bp to 23 bp, comprising 116 bp in total; the longest spacer region falls between trnS2 and ND1.

**Table 2.** Organization of the mitochondrial genome of *Pentatoma rufipes*.

Gene	Strand	Position	Anticodon	Size(bp)	Start codon	Stop codon	Intergenetic nucleotides*
trnI	J	1–67	GAT	67			
trnQ	N	65–134	TTG	70			–3
trnM	J	137–205	CAT	69			2
ND2	J	206–1189		984	ATT	TAA	0
trnW	J	1198–1265	TCA	68			8
trnC	N	1258–1321	GCA	64			–8
trnY	N	1331–1397	GTA	67			9
COX1	J	1407–2943		1537	TTG	T	9
trnL2*^UUR^*	J	2944–3008	TAA	65			0
COX2	J	3009–3687		679	ATA	T	0
trnK	J	3688–3761	CTT	74			0
trnD	J	3761–3822	GTC	62			–1
ATP8	J	3823–3981		159	TTG	TAA	0
ATP6	J	3975–4649		675	ATG	TAA	–7
COX3	J	4652–5440		789	ATG	TAA	2
trnG	J	5446–5510	TCC	65			5
ND3	J	5511–5864		354	ATC	TAA	0
trnA	J	5873–5943	TGC	71			8
trnR	J	5960–6024	TCG	65			16
trnN	J	6033–6101	GTT	69			8
trnS1*^AGN^*	J	6101–6170	ACT	70			–1
trnE	J	6171–6238	TTC	68			0
trnF	N	6237–6301	GAA	65			–2
ND5	N	6301–8007		1707	ATT	TAA	–1
trnH	N	8009–8076	GTG	68			1
ND4	N	8079–9410		1332	ATG	TAA	2
ND4L	N	9404–9691		288	ATT	TAA	–7
trnT	J	9694–9758	TGT	65			2
trnP	N	9759–9820	TGG	62			0
ND6	J	9823–10299		477	ATG	TAA	2
CYTB	J	10304–11440		1137	ATG	TAA	4
trnS2*^UCN^*	J	11456–11524	TGA	69			15
ND1	N	11548–12477		930	ATA	TAA	23
trnL1*^CUN^*	N	12472–12539	TAG	68			–6
16S rRNA	N	12540–13816		1277			0
trnV	N	13817–13886	TAC	70			0
12S rRNA	N	13887–14707		821			0
CR		14708–15887		1180			0

^*^ Numbers correspond to nucleotides separating a gene from an upstream one; negative numbers indicate that adjacent cent genes overlap.

### Nucleotide composition and codon usage

The base content and skewness of the genes in the *P. rufipes* mitogenome is shown in Table 3. The base composition of the entire sequence is in the order of A(42.0%)>T(35.7%)>C(12.4%)>G(9.9%), with a bias toward A + T. This bias was observed in all genetic elements, with an A + T content of 77.1% in PCGs, 77.7% in tRNAs, 79.8% in rRNAs, and 78.7% in the control region. The complete genome also shows a clear AC-skew (AT-skew = 0.08, GC-skew = −0.11), indicating a greater abundance of A than T and of C than G.

**Table 3.** Nucleotide composition and skewness of the *Pentatoma rufipes* mitochondrial genome.

Feature	Length(bp)	A%	C%	G%	T%	A+T%	AT-skew	GC-skew
Whole genome	15737	42.0	12.4	9.9	35.7	77.7	0.08	–0.11
PCGs	11046	34.2	11.1	11.8	42.9	77.1	–0.11	0.03
PCG-J	6800	37.2	12.6	11.7	38.5	75.7	–0.02	–0.04
PCG-N	4246	29.4	8.8	11.9	49.9	79.3	–0.26	0.15
tRNA genes	1460	39.7	10.0	12.3	38.0	77.7	0.02	0.10
tRNA genes-J	936	40.6	11.0	11.1	37.3	77.9	0.04	0.01
tRNA genes-N	524	38.0	8.2	14.4	39.3	77.3	–0.02	0.27
rRNA genes	2053	35.6	7.6	12.6	44.2	79.8	–0.11	0.25
Control region	1142	38.3	13.6	7.6	40.4	78.7	–0.03	–0.28

**Figure 2.**
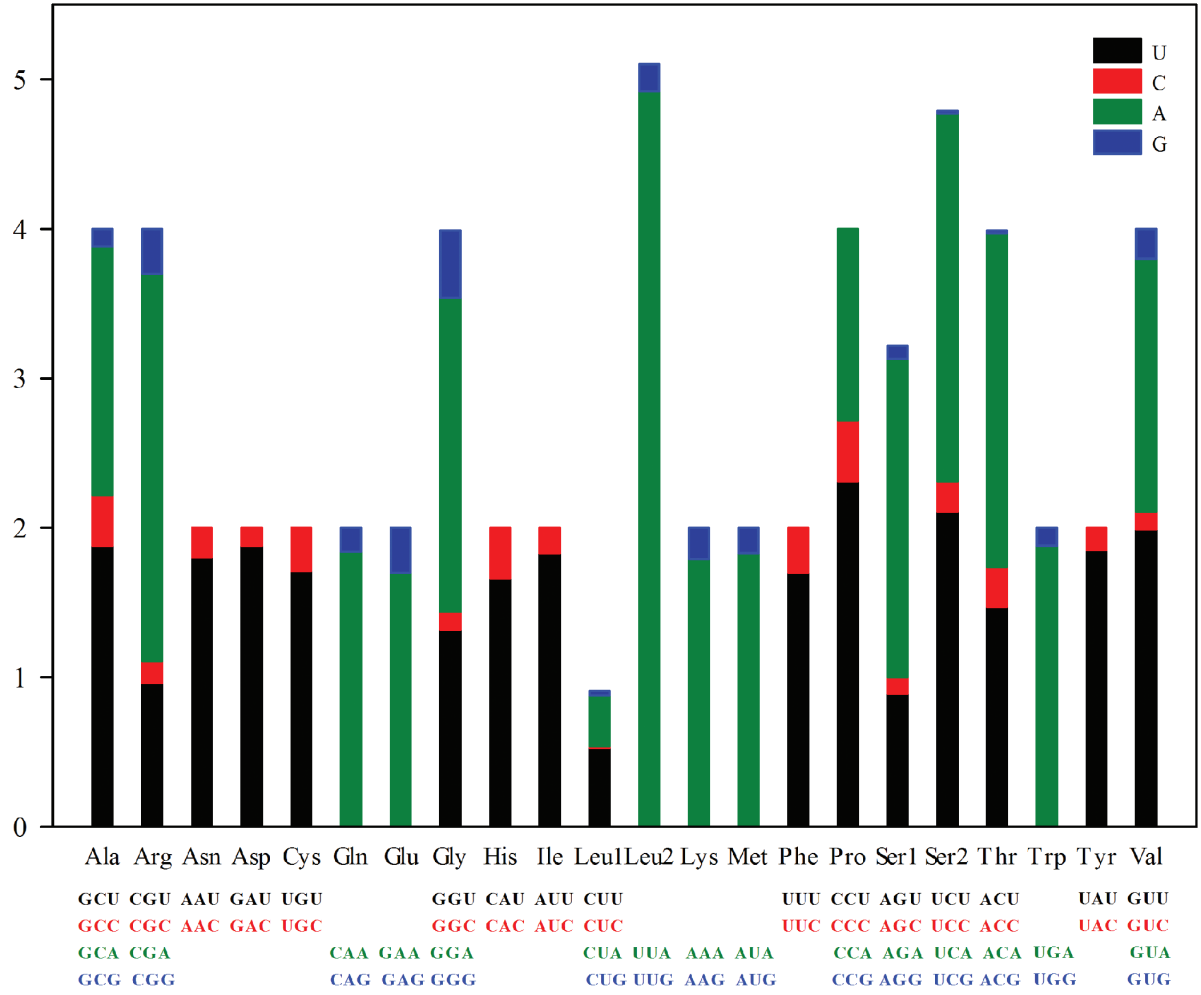
The relative synonymous codon usage (RSCU) in the mitogenome of *Pentatoma rufipes*.

**Figure 3.**
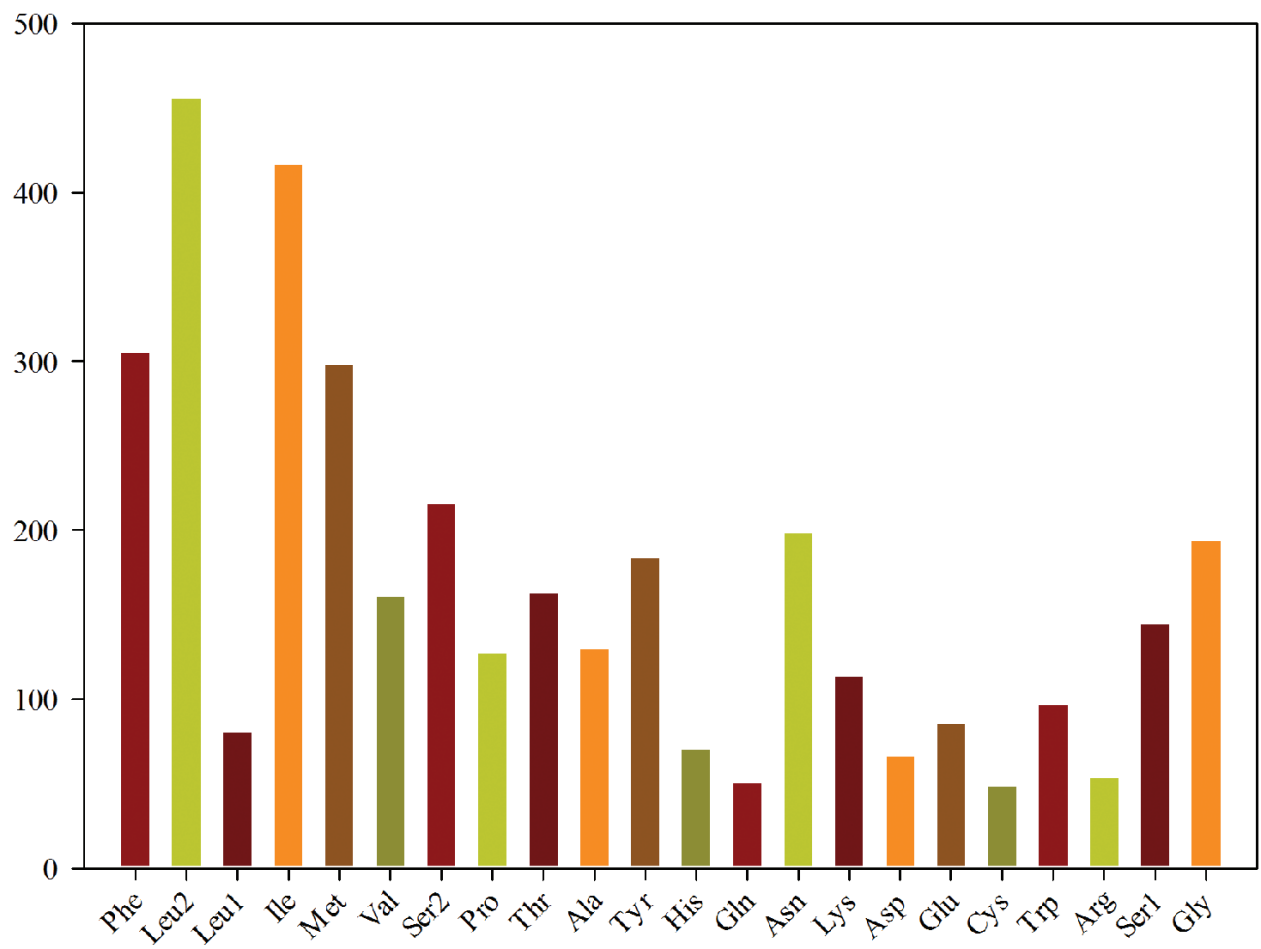
Amino acid composition in the *Pentatoma rufipes* mitogenome. Codon families are provided on the x-axis. Numbers of codons of each amino acid are provided on the y-axis.

The preference for nucleotide composition is also reflected in codon use. The relative synonymous codon usage values for the *P. rufipes* mitogenome are summarized in Figure 2 and Table 4. Figure 3 shows the amino acid composition of the *P. rufipes* mitogenome. The most common amino acids are Phe, Leu, Ile, and Met, and their most abundant codons (UUU for Phe, UUA for Leu2, AUU for Ile, and AUA for Met) are all composed of A and/or T. For each amino acid, the most commonly used coded codons are NNA and NNU, reflecting the skew of the nucleotide composition toward AT. In addition, the most frequently used codons do not strictly correspond to the tRNA anticodons for most amino acids.

**Table 4.** Codon number and RSCU in the *Pentatoma rufipes* mitochondrial PCGs.

Amino acid	Codon	N	RSCU	N+	RSCU+	N–	RSCU–
Phe	UUU	**260**	1.7	118	1.49	**142**	1.92
	UUC	46	0.3	40	0.51	6	0.08
Leu2	UUA	**440**	4.92	**238**	4.89	202	4.95
	UUG	16	0.18	6	0.12	10	0.24
Leu1	CUU	47	0.53	19	0.39	28	0.69
	CUC	1	0.01	1	0.02	0	0
	CUA	30	0.34	26	0.53	4	0.1
	CUG	3	0.03	2	0.04	1	0.02
Ile	AUU	**382**	1.83	**255**	1.8	127	1.91
	AUC	35	0.17	29	0.2	6	0.09
Met	AUA	**274**	1.83	**179**	1.86	95	1.78
	AUG	25	0.17	13	0.14	12	0.22
Val	GUU	80	1.99	33	1.43	47	2.72
	GUC	5	0.12	1	0.04	4	0.23
	GUA	68	1.69	51	2.22	17	0.99
	GUG	8	0.2	7	0.3	1	0.06
Ser2	UCU	95	2.11	31	1.24	64	3.18
	UCC	9	0.2	6	0.24	3	0.15
	UCA	111	2.46	76	3.04	35	1.74
	UCG	1	0.02	0	0	1	0.05
Pro	CCU	74	2.31	48	2.04	26	3.06
	CCC	13	0.41	9	0.38	4	0.47
	CCA	41	1.28	37	1.57	4	0.47
	CCG	0	0	0	0	0	0
Thr	ACU	60	1.47	42	1.33	18	1.95
	ACC	11	0.27	5	0.16	6	0.65
	ACA	91	2.23	78	2.48	13	1.41
	ACG	1	0.02	1	0.03	0	0
Ala	GCU	61	1.88	42	1.77	19	2.17
	GCC	11	0.34	9	0.38	2	0.23
	GCA	54	1.66	44	1.85	10	1.14
	GCG	4	0.12	0	0	4	0.46
Tyr	UAU	170	1.85	67	1.7	103	1.96
	UAC	14	0.15	12	0.3	2	0.04
His	CAU	59	1.66	45	1.58	14	2
	CAC	12	0.34	12	0.42	0	0
Gln	CAA	47	1.84	35	2	12	1.5
	CAG	4	0.16	0	0	4	0.5
Asn	AAU	179	1.8	114	1.74	65	1.91
	AAC	20	0.2	17	0.26	3	0.09
Lys	AAA	102	1.79	73	1.9	29	1.57
	AAG	12	0.21	4	0.19	8	0.43
Asp	GAU	63	1.88	38	1.81	25	2
	GAC	4	0.12	4	0.19	0	0
Glu	GAA	73	1.7	56	1.9	17	1.26
	GAG	13	0.3	3	0.1	10	0.74
Cys	UGU	42	1.71	12	1.6	30	1.76
	UGC	7	0.29	3	0.4	4	0.24
Trp	UGA	91	1.88	68	1.97	23	1.64
	UGG	6	0.12	1	0.03	5	0.36
Arg	CGU	13	0.96	2	0.23	11	2.32
	CGC	2	0.15	1	0.11	1	0.21
	CGA	35	2.59	30	3.43	5	1.05
	CGG	4	0.3	2	0.23	2	0.42
Ser1	AGU	40	0.89	14	0.56	26	1.29
	AGC	5	0.11	3	0.12	2	0.1
	AGA	96	2.13	69	2.76	27	1.34
	AGG	4	0.09	1	0.04	3	0.15
Gly	GGU	64	1.32	28	0.9	36	2.06
	GGC	6	0.12	2	0.06	4	0.23
	GGA	102	2.1	82	2.65	20	1.14
	GGG	22	0.45	12	0.39	10	0.57

N, N+, and N– are respectively the number of codons used in the total protein codon gene, the majority strand protein codon gene (J-strand), and the minority strand protein codon gene (N-strand). Values in bold type stand for most commonly used codon for the amino acid. Underlined codons stand for the cognate codon of tRNA for each amino acid.

### PCG regions

Most *P. rufipes*PCGs share the ATN start codon (five with ATG, three with ATT, two with ATA, and one with ATC), except for COX1 and ATP8, which start with TTG. COX1 and COX2 sequences terminate with a single T, and the stop codon for the remaining genes is TAA. The AT content (77.1%) of the 13 PCGs exceeded the GC content (22.9%), and the AT bias is moderately negative (absolute value: 0.1–0.2).

**Figure 4.**
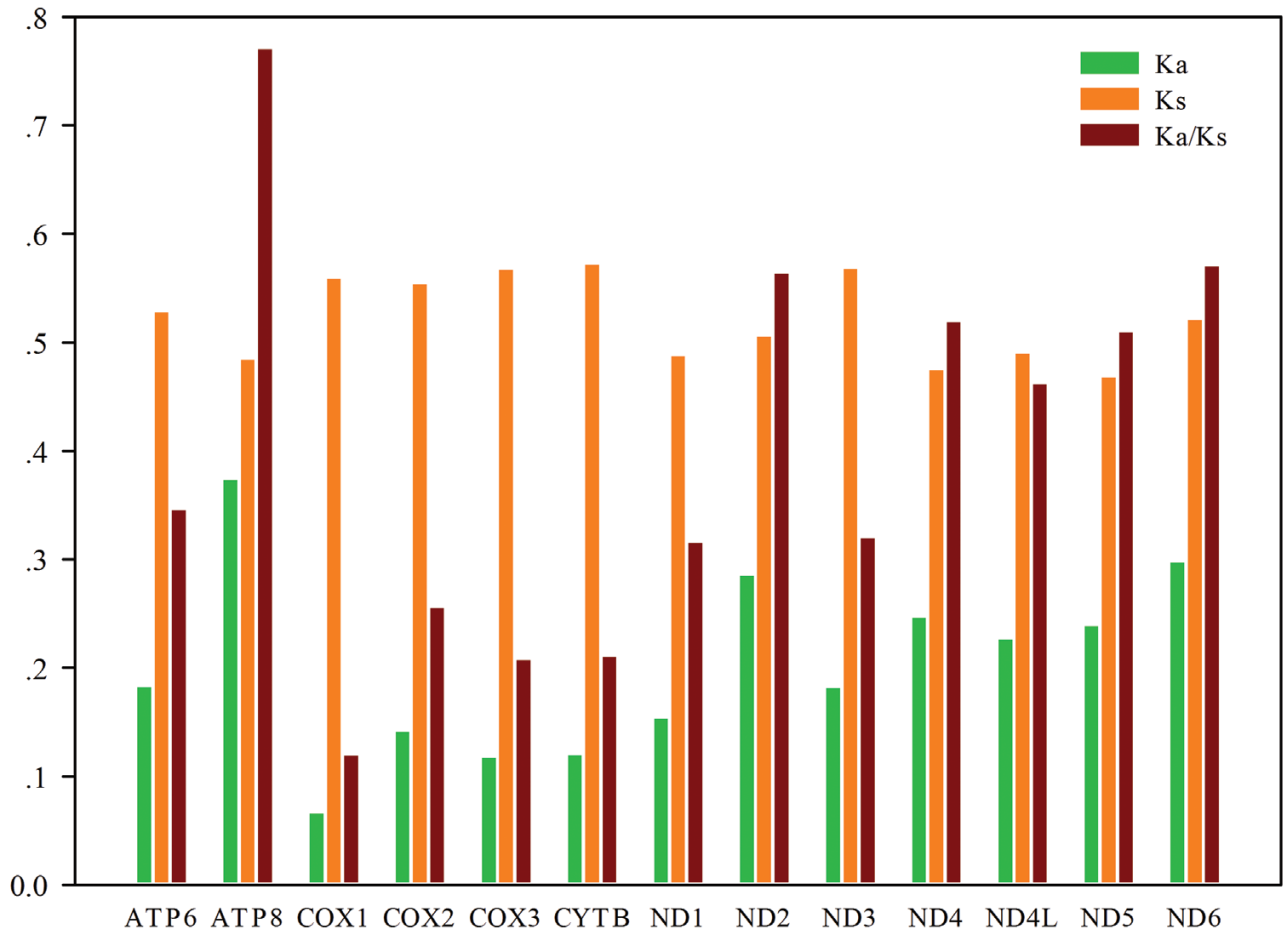
Evolutionary rates of 13 PCGs in Pentatomoidea. Rate of non-synonymous substitutions (Ka), rate of synonymous substitutions (Ks) and ratio of rate of non-synonymous substitutions to rate of synonymous substitutions (Ka/Ks) are calculated for each PCG.

In addition, we calculated the synonymous substitutions (Ks), non-synonymous substitutions (Ka), and the Ka/Ks ratios of the 13 PCGs from Pentatomoid insects. We also compared the evolutionary rates of the 13 PCGs (Fig. 4). The evolutionary rate of ATP8 was the fastest, followed by that of ND6, and the COX1 gene was the most conservative with the slowest rate. The evolutionary rates of the other genes were in the order of ND2 > ND4 > ND5 > ND4L > ATP6 > ND3 > ND1 > COX2 > COX3 > CYTB. Moreover, the Ks values of the 13 PCGs were all greater than the Ka values, and the Ka/Ks ratio was <1, which indicates that the genes are subject to purifying selection.

### tRNA genes, rRNA genes, and the control region

**Figure 5.**
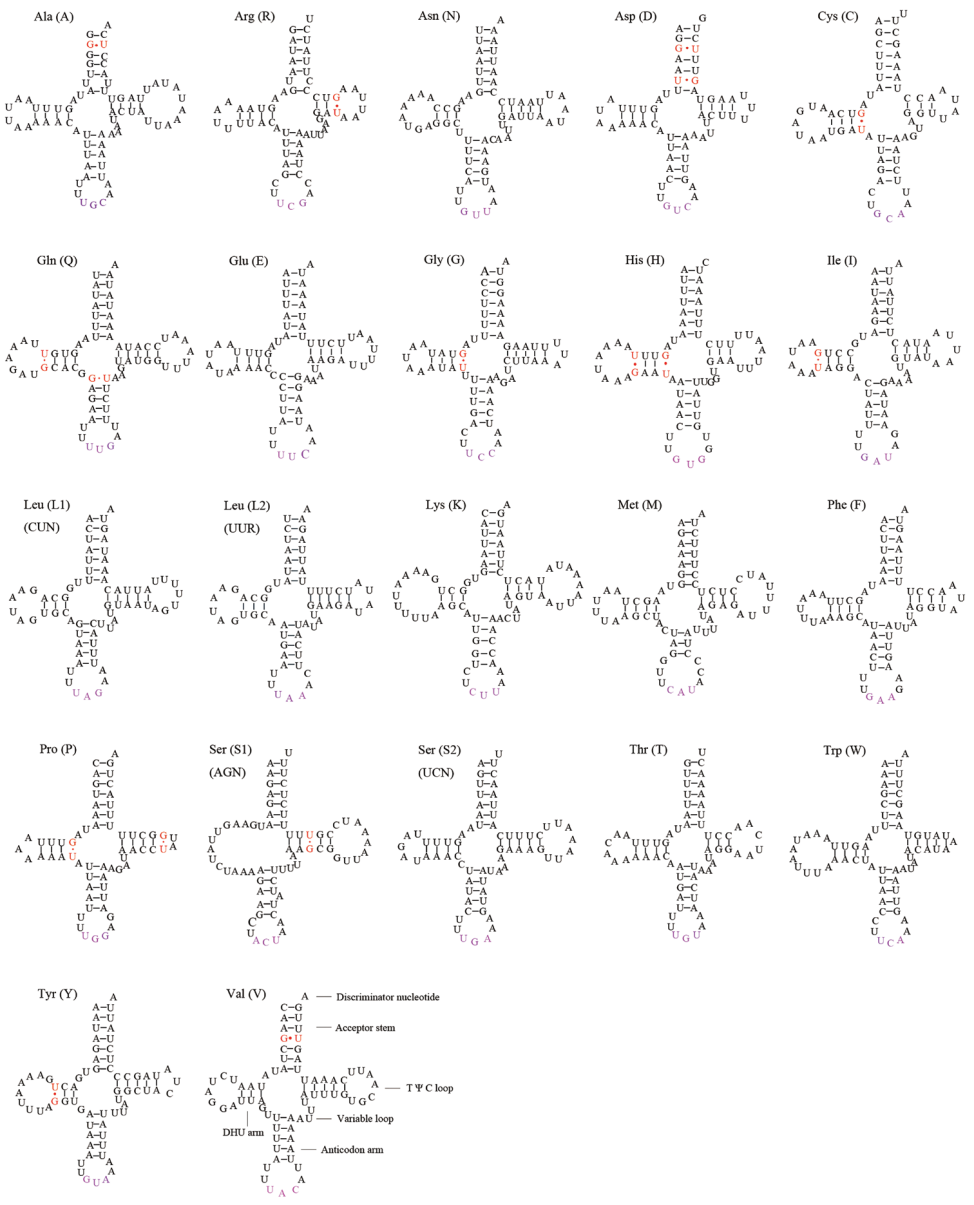
Predicted secondary structure of tRNA genes in the *Pentatoma rufipes* mitogenome.

We detected 22 tRNA genes, which can transport all 20 amino acids, in the mitogenome of *P. rufipes*. There are two tRNAs each for leucine and serine: trnL1 (CUN) and trnL2 (UUR), and trnS1 (AGN) and trnS2 (UCN), respectively. The anticodons of trnL are TAA and TAG, and the anticodons of trnS are ACT and TGA. The 22 tRNA genes span 1,481 bp, between 62 and 74 bp in length. Although trnS1 lacks a dihydrouridine arm, the other tRNA genes all have the classic clover leaf secondary structure. In addition to the typical base pairs (A-U and G-C), some wobble G-U pairs appear in these secondary structures, which can form stable chemical bonds between G and U; In addition, atypical pairing of U-U and U-C is also found (Fig. 5).

**Figure 6.**
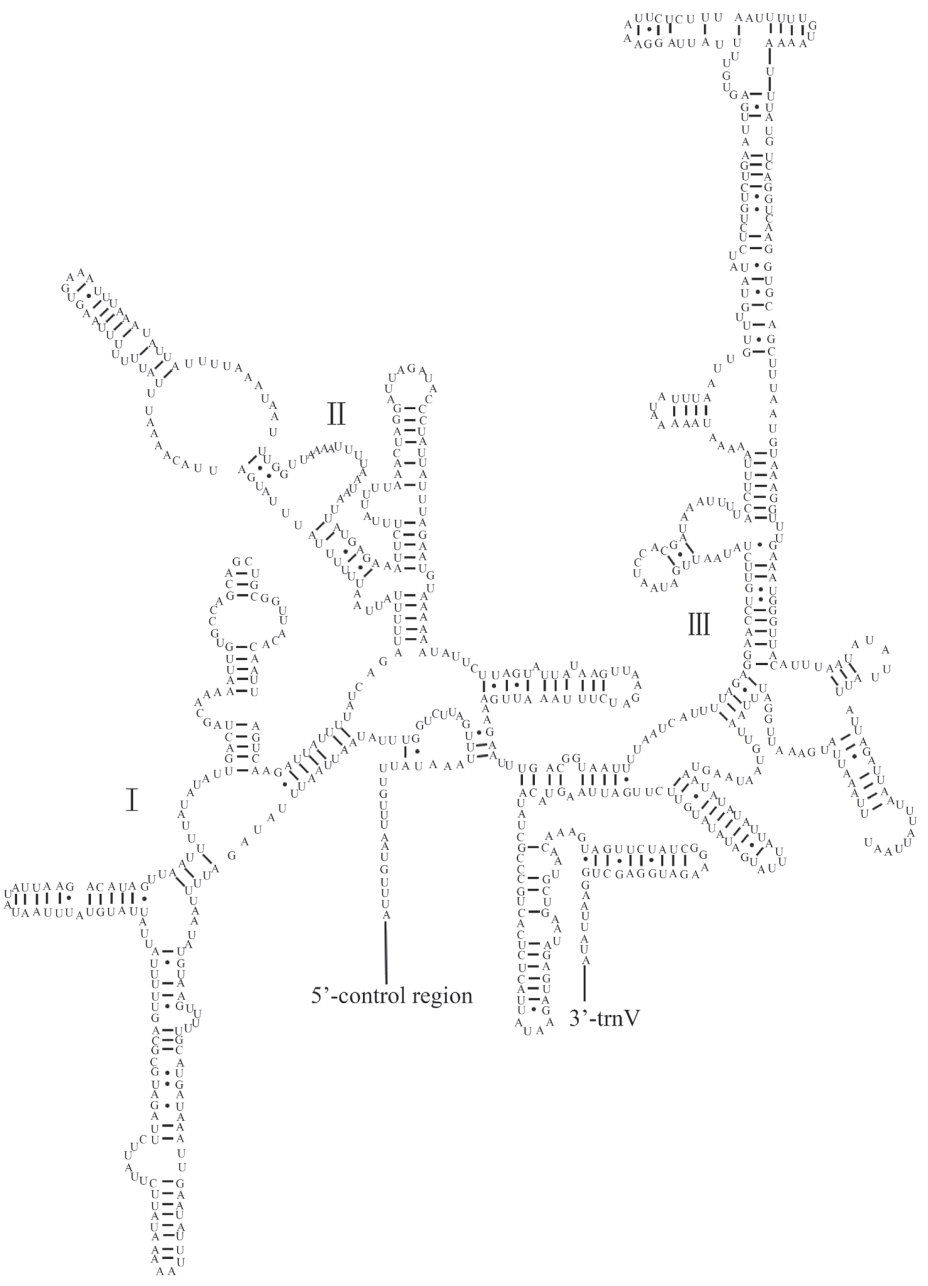
Predicted secondary structure of the 12S rRNA in the *Pentatoma rufipes* mitogenome.

**Figure 7.**
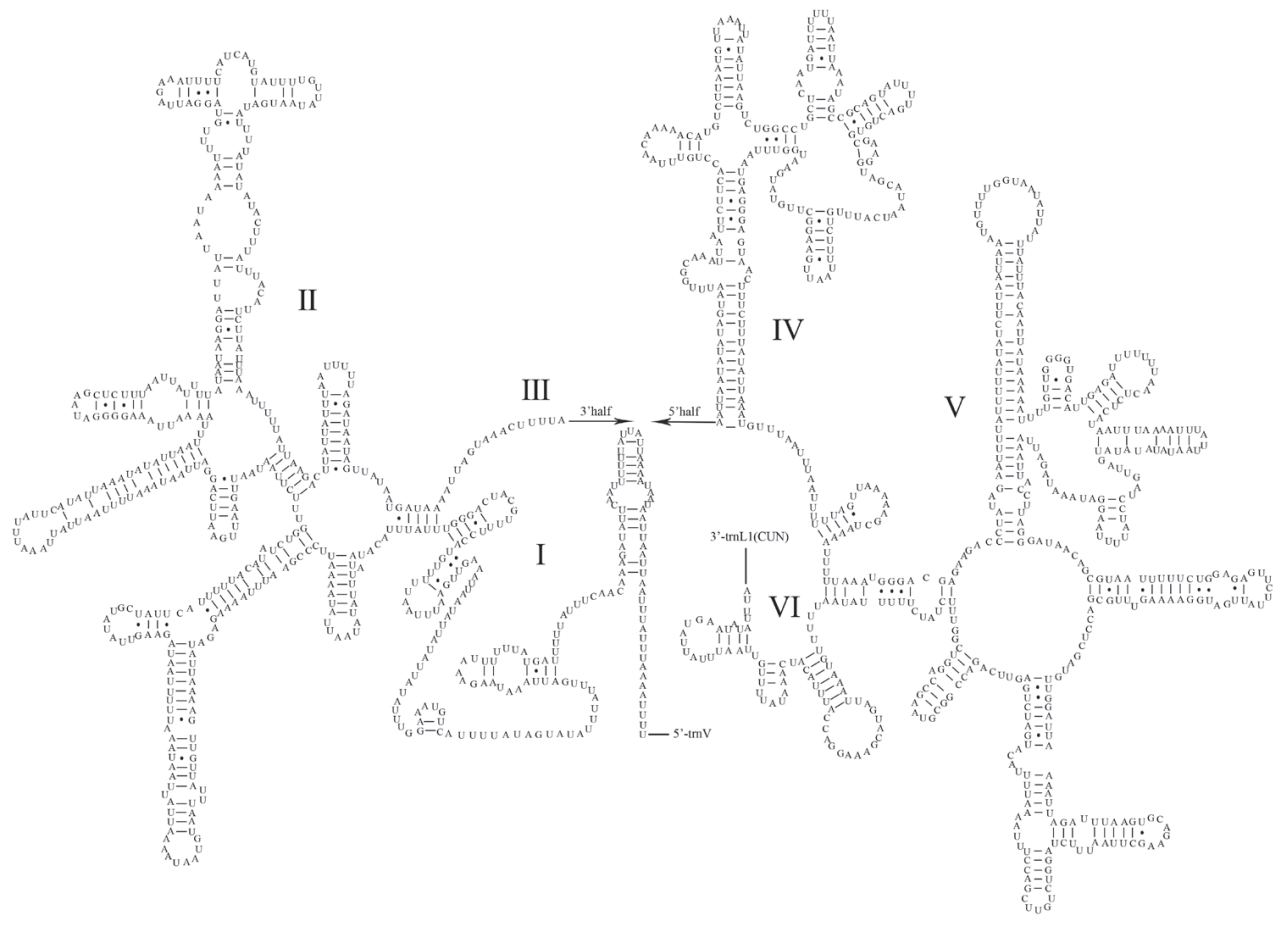
Predicted secondary structure of the 16S rRNA in the *Pentatoma rufipes* mitogenome.

The two *P. rufipes*rRNA genes (12S rRNA and 16S rRNA) are encoded on the N-strand. The 16S rRNA gene is located between trnL1 (CUN) and trnV, which is 1,277 bp in length, and there is no gene overlap between 16S rRNA and the two tRNA genes. The 12S rRNA gene (821 bp) is located between trnV and the control region, similar to the published pentatomid mitogenomes. The base content of the rRNA genes is in the order of T (44.2%) > A (35.6%) > G (12.6%) > C (7.6%). The AT-skews are negative, and the GC-skews are positive. The complete secondary structures of the 12S rRNA and 16S rRNA genes are shown in Figures 6, 7, respectively.

The control region of the mitogenome of *P. rufipes* is located between the 12S rRNA gene and trnI. The control region is 1,180 bp long, making it the longest noncoding region in the mitogenome, and has an A + T content of 78.7%. The AT-skew and GC-skew in the control area are –0.03 and –0.28, respectively, indicating that the content of T is higher than that of A and the content of C is higher than that of G.

### Saturation test

**Figure 8.**
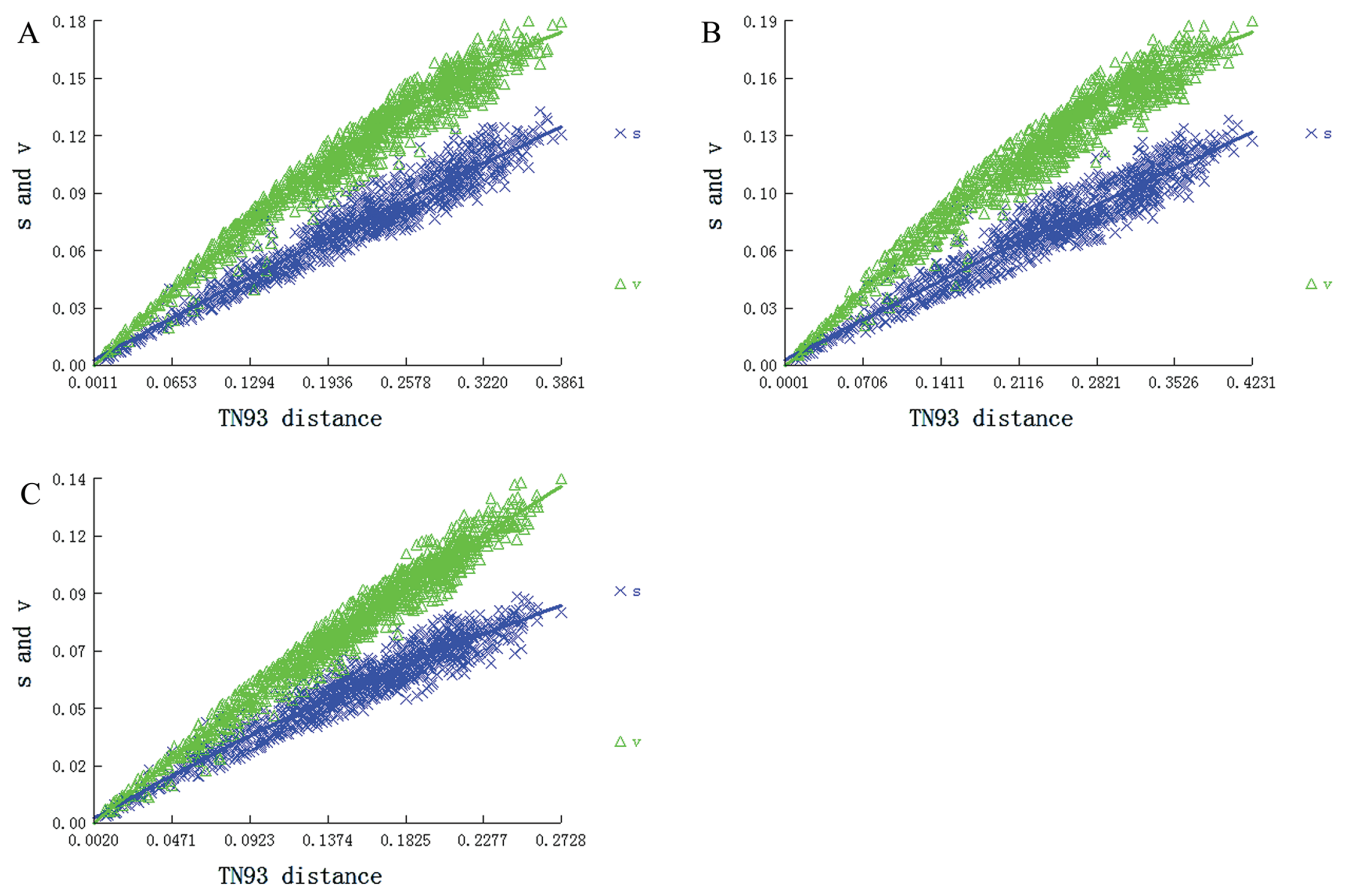
Substitution patterns of PCGRNA, PCG and PCG12 matrices. The graphs represent the increase in TN93 distance **A** PCGRNA saturation plot **B** PCG saturation plot **C** PCG12 saturation plot.

To eliminate the negative effect of the substitution saturation in the phylogenetic analysis, saturation tests on the three data sets were conducted. Nucleotide sequence substitution saturation is usually determined by analyzing the relationship between the transition and transversion values against the corresponding corrected genetic distance. In all tests, the Xia saturation index (Iss) was below the critical values for a symmetric (Iss.cSym) and asymmetric (Iss.cAsym) topology (Fig. 8). The values for base transition and transversion were linearly associated with the corrected genetic distance, indicating that the nucleotide sequences of these three datasets were not saturated, making them suitable for constructing phylogenetic trees.

### Phylogenetic analyses

**Figure 9.**
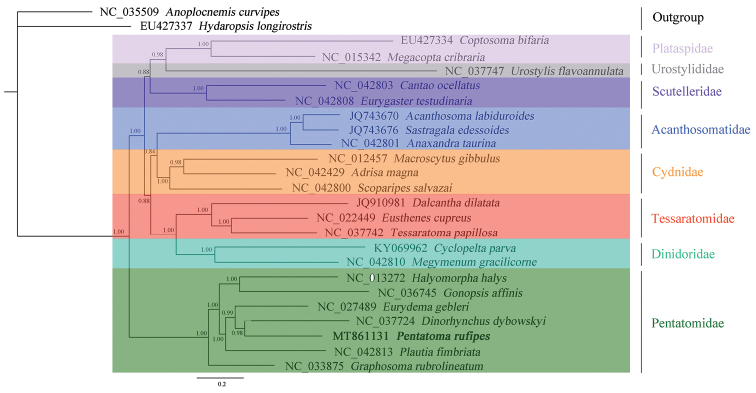
Phylogenetic tree inferred from PCGRNA constructed using BI analysis. The number on the branches indicates Bayesian posterior probabilities.

**Figure 10.**
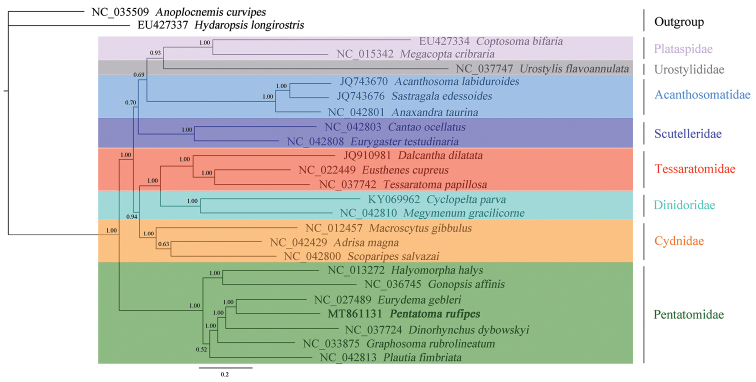
Phylogenetic tree inferred from PCG constructed using BI analysis. The number on the branches indicates Bayesian posterior probabilities.

**Figure 11.**
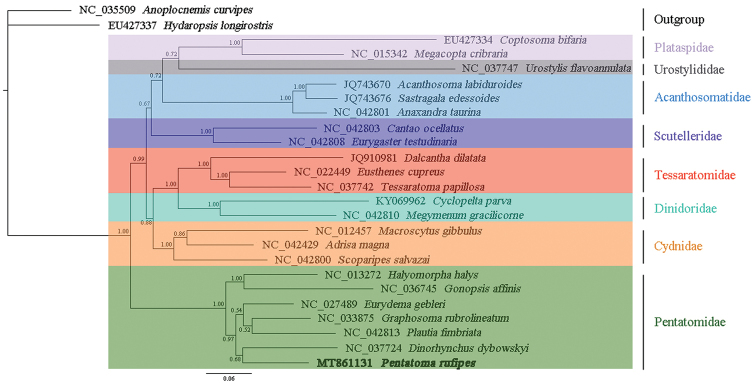
Phylogenetic tree inferred from PCG12 constructed using BI analysis. The number on the branches indicates Bayesian posterior probabilities.

We reconstructed the phylogenetic trees of eight families in Pentatomoidea from three datasets (PCGRNA, PCG, and PCG12) using Bayesian inference method. The topological structures of the trees were similar, especially PCG and PCG12 showed similar family-level relationships (Figs 9–11). Among the three trees, the Bayesian posterior probability value of the phylogenetic tree based on the PCGRNA dataset was the highest. Phylogenetic analysis based on PCGRNA data showed that *P. rufipes* and *Dinorhynchus dybowskyi* Jakovlev were closely related, these two species formed sister groups with *E. gebleri*, and *P. rufipes* and *Graphosoma rubrolineatum* (Westwood) had the farthest relationship. However, the results in the phylogenetic analysis based on PCG data were somewhat different from the above. In this analysis, *P. rufipes* and *E. gebleri* were the most closely related species, and they were sister groups with *D. dybowskyi*.

## Discussion and conclusions

In this study, we sequenced the complete mitogenome of *P. rufipes* using NGS technology, revealing a mitogenome that is 15,887-bp-long containing 37 genes. The order of the 37 genes is consistent with other published mitogenome of Hemiptera (Hua et al. 2009; Zhang et al. 2018; Zhao et al. 2019). There are three obvious overlapping regions in mitochondrial genome of *P. rufipes*. The longest overlap located between trnW and trnC, which is 8 bp in length, and the overlap bases are AAGCTTTA. This overlap also showed in most species of Pentatomidae (Yuan et al. 2015a and Zhao et al. 2019). The other two pairs of genes, namely ATP8/ATP6 and ND4/ND4L, overlap by 7 bp, and both overlap bases are ATGATAA, which is consistent with other hemipteran insects (Zhang et al. 2019; Zhao et al. 2020). The longest spacer region falls between trnS2 and ND1, which is consistent with the findings of other studies (Hua et al. 2008; Zhao et al. 2019). The difference of mitogenome size between *P. rufipes* and other species of Hemiptera due to the length difference of the noncoding region.

The composition of the four bases in the *P. rufipes* mitogenome suggested highly unbalanced (A>T>C>G). The nucleotide composition shows an obvious AT preference, and the entire genome shows AT-skew and CG-skew. The above characteristics of mitogenome base composition of *P. rufipes* are ubiquitous to all sequenced species of Pentatomidae. The preference of bases composition is generally considered to be caused by asymmetric mutation and selection pressure of the four bases (Brown et al. 2005). Consistent with most species of Hemiptera, the PCGs of this species use the common triplet codon ATN as the start codon, TAA and a single T as the stop codon (Hua et al. 2008; Zhao et al. 2019).

The secondary structures of tRNAs for *P. rufipes* is conserved and trnS1 lacks DHU arm, these features meet the character of metazoan mitochondrial genomes (Wolstenholme 1992). In addition to the typical Watson-Crick pairing (G-C and A-U), there are also some typical pairings such as U-G, U-C and U-U. Some scholars have proposed that those tRNAs with non-Watson-Crick matches can be transformed into fully functional proteins through post-transcriptional mechanisms (Chao et al. 2008; Pons et al. 2014). The rRNA secondary structure of this species is also conserved. The 12S rRNA sequence includes three domains and the 16S rRNA sequence includes six domains (domain III is absent), which is similar to pentatomoid insects.

The phylogenetic result suggested that there are some different topology compared to other studies, but we infer that the possible reasons are as follows: first, the number and taxon of samples selected are different. In this study, the phylogenetic relationship between *Pentatoma* and *Plautia* was relatively close, and they were far from *Graphosoma*. However, when the phylogenetic tree was constructed with *Pentatoma semiannulata*, the relationship between *Pentatoma* and *Graphosoma* was closer (Wang et al. 2021). Second, the selection of outgroup also affects the topological structure of phylogenetic tree. Comparing our results with Zhao et al. (2017) and Liu et al. (2019), because of the three studies choose different species as the outgroup, we got different phylogenies. Third, different molecular markers also might affect phylogenetic relationships. Grazia et al. (2008) supported the monophyly of Pentatomoidea and most of the included families based on morphological characters and molecular markers (16S rRNA, 18S rRNA, 28S rRNA, and COI). Lis et al. (2012) constructed similar phylogenetic trees to our study using 12S and 16S rDNA datasets. Tian et al. (2011) (based on Hox genes), Liu et al. (2019) (based on PCGRNA and PCG12RNA) and Li et al. (2005) (based on 18S rDNA and COX1 sequence) also put forward their own opinions on the phylogenetic relationship of the superfamily. Our three topologies revealed that the Bayesian posterior probability of the tree based on PCGRNA sequences was significantly higher than that of the trees based on the PCG data, indicating that inclusion of tRNA and rRNA genes improves the accuracy of the analysis, which is consistent with the findings of the study conducted by Cameron et al. (2007, 2009).

In summary, the mitogenome of *P. rufipes* has typical sequence structures, and the gene content, nucleotide composition, codon usage, RNA structures, and rates of PCGs evolution are similar to those of other published pentatomid genomes. The mitochondrial genome of *P. rufipes* reveals the phylogenetic location of *Pentatoma*, indicating that the mitogenome can be used to reveal phylogenetic relationships among different taxonomic levels of insects. However, more insect mitogenomes should be sequenced, which would provide more insight into the phylogenetic relationships of species from different taxa.
